# Prevalence and predictors of aortic root abscess among patients with left-sided infective endocarditis: a cross-sectional comparative study

**DOI:** 10.1186/s43044-020-00098-6

**Published:** 2020-09-29

**Authors:** Kareem Mahmoud, Tarek Hammouda, Hossam Kandil, Marwa Mashaal

**Affiliations:** Cairo University, Cairo, Egypt

**Keywords:** Aortic root abscess, Infective endocarditis, Paravalvular leakage

## Abstract

**Background:**

Aortic root abscess (ARA) is a major complication of infective endocarditis that is associated with increased morbidity and mortality. Limited data are present about patient characteristics and outcomes in this lethal disease. We aimed to study the clinical and echocardiographic characteristics of patients with ARA compared to patients with left-sided infective endocarditis without ARA. We included patients with a definite diagnosis of left-sided infective endocarditis according to modified Duke’s criteria. The patients were classified into two groups according to the presence of ARA (ARA and NO-ARA groups). All the patients were studied regarding their demographic data, clinical characteristics, laboratory and imaging data, and complications.

**Results:**

We included 285 patients with left-sided infective endocarditis. The incidence of ARA was 21.4% (61 patients). Underlying heart disease, mechanical prosthesis, bicuspid aortic valve, and prior IE were significantly higher in ARA. The level of CRP was higher in ARA (*p* = 0.03). ARA group showed more aortic valve vegetations (73.8% vs. 37.1%, *p* < 0.001), more aortic paravalvular leakage (26.7% vs. 4.5%, *p* < 0.001), and less mitral valve vegetations (21.3% vs. 68.8%, *p* < 0.001). Logistic regression analysis showed that the odds of ARA increased in the following conditions: aortic paravalvular leak (OR 3.9, 95% CI 1.2–13, *p* = 0.03), mechanical prosthesis (OR 3.6, 95% CI 1.5–8.7, *p* = 0.005), aortic valve vegetations (OR 3.0, 95% CI 1.2–8.0, *p* = 0.02), and undetected organism (OR 2.3, 95% CI 1.1–4.6, *p* = 0.02), while the odds of ARA decreased with mitral valve vegetations (OR 0.2, 95% CI 0.08–0.5, *p* = 0.001). We did not find a difference between both groups regarding the incidence of major complications, including in-hospital mortality.

**Conclusion:**

In our study, ARA occurred in one fifth of patients with left-sided IE. Patients with mechanical prosthesis, aortic paravalvular leakage, aortic vegetations, and undetected organisms had higher odds of ARA, while patients with mitral vegetations had lower odds of ARA.

## Background

Infective endocarditis (IE) is a deadly disease with high mortality rates and high rates of complications [1]. Given the relatively low incidence of the disease, until recently, guidelines relied on expert opinion rather than controlled trials [2]. The majority of studies in the field of infective endocarditis were observational, with a limited number of randomized-controlled trials and meta-analyses [3–5].

Periannular extension of the infection can occur, causing periannular abscesses. Aortic root abscess has been found in up to 46% of aortic valve IE cases [6]. The incidence of infective endocarditis and aortic root abscess usually vary among different countries. Antibiotics alone may occasionally sterilize an abscess cavity. However, without surgical intervention, many patients die of congestive heart failure, sepsis, or both. Moreover, taking a full course of the appropriate antibiotic to achieve a healed status is not always possible, exposing patients to the risk of an aggressive surgical approach during the active phase [7, 8].

Few case series described the worldwide experience in such complication, pointing out the possible predictors, clinical course, and results of various management approaches [6, 9]. Our study aimed to determine the prevalence, clinical and echocardiographic predictors of aortic root abscess in patients with left-sided infective endocarditis.

## Methods

### Subjects

This study was a cross-sectional comparative study that included patients with a definite diagnosis of left-sided infective endocarditis according to modified Duke’s criteria [10]. The patients were recruited as part of the infective endocarditis registry from January 2014 to February 2019. The infective endocarditis team consists of cardiologists, cardiothoracic surgeons, echocardiographers, microbiologists, and pathologists. We obtained written informed consent from all patients enrolled in the study to collect their data. The institutional ethics review committee approved the study.

We included 285 patients with left-sided infective endocarditis and divided them into two groups:

*Aortic root abscess (ARA) group*: included patients with left-sided infective endocarditis complicated with aortic root abscess (61 patients).

*No aortic root abscess (NO-ARA) group*: included patients with left-sided infective endocarditis not complicated with aortic root abscess (225 patients).

### Methods

All study population had the following diagnostic workup:

*History*: age, gender, underlying cardiac conditions, comorbidities (e.g., hypertension, diabetes mellitus, liver and kidney diseases, autoimmune disease, and chronic steroid use), symptoms related to infective endocarditis, and its duration.

*Clinical examination*: vital signs, signs of heart failure, and findings related to infective endocarditis (e.g., clubbing, Roth spot, splenomegaly, neurological affection, and cutaneous manifestations).

*Laboratory workup*: hemoglobin, total leukocytic count, serum creatinine, and CRP.

*Blood culture and sensitivity* according to the recent guidelines [11].

*Serology* for Aspergillus, Brucella, Coxiella, Listeria, Bartonella.

NB. We reported the causative microorganisms according to the results of blood cultures or serology. The undetected organism was the condition when both blood cultures and serology were negative.

*Transthoracic echocardiography (TTE)* was done for all patients as an initial investigation in cases of infective endocarditis within 24–48 h of admission [12].

**Fig. 1:**
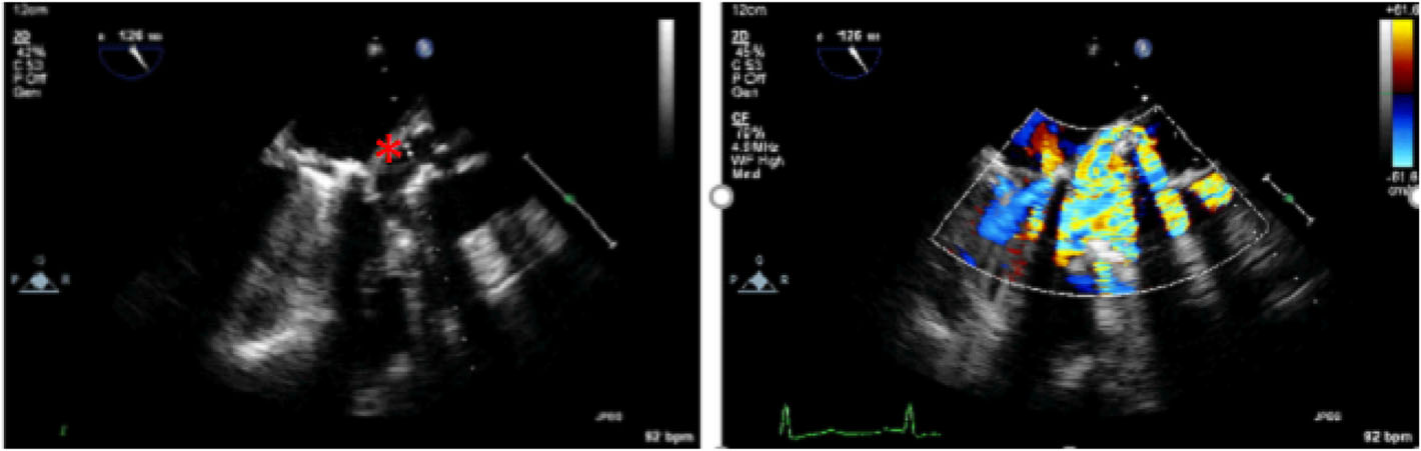
TEE showing large aortic root abscess (ARA). The left image: ARA is seen at the posterior aspect of the aortic root as marked by *. The right image: considerable paravalvular leakage from the aorta to the left ventricular outflow tract

*Transesophageal echocardiography (TEE)* was done for the diagnosis of IE and the detection of local complications. Images were obtained using the Philips X7-2t ultrasound probe on the Philips IE33 (Philips Medical Systems, Andover, Massachusetts). Sedation was done using IV midazolam, given in an increment of 1–2 mg. The patients were asked to fast for 6 h. Standard mid-esophageal, transgastric, and upper esophageal views were obtained as recommended by the American Society of Echocardiography guidelines for performing TEE examination [13]. Vegetation was defined as an oscillating or non-oscillating intracardiac mass on a valve or other endocardial structures. Abscess was identified as a thickened, non-homogeneous echodense or echolucent perivalvular space [12, 14]. Figure 1 shows an example of the use of TEE in diagnosing ARA.

*Abdominal ultrasonography* was performed for all patients to evaluate the complications of infective endocarditis, as splenic infarctions and splenic abscess.

*Duplex ultrasound or CT angiography*: Either of these tests was done on clinical suspicion of peripheral embolization (e.g., symptoms of limb ischemia, absent pulsations).

*CT brain, MRI brain, or CT cerebral angiography*: Either of these tests was done for almost all cases as a standard practice as long as it is not interfering with the urgency of the surgery. This enabled us to detect intracranial complications, especially silent mycotic aneurysms.

In-hospital clinical major complications were defined as the presence of one of the following:

- In-hospital mortality
- Any clinically overt central nervous system event (embolic brain infarction, brain hemorrhage, transient ischemic attack, or meningitis)
- Peripheral embolization to extremities or abdominal viscera
- Congestive heart failure NYHA classes III–IV

Sepsis: defined as life-threatening organ dysfunction caused by a dysregulated host response to infection with the following clinical criteria: suspected or documented infection and an acute increase of two or more SOFA (Sepsis-related Organ Failure Assessment) score points [15].

Acute kidney injury: defined as either a rise in serum creatinine by 0.3 mg/dL or more within 48 h, a rise in serum creatinine to 1.5 times baseline or more within the last 7 days, or a decline in urine output to less than 0.5 mL/kg/h for 6 h [16].

### Primary objectives

To estimate the prevalence of aortic root abscess in patients with left-sided IE.

To assess the association between the clinical and echocardiographic data mentioned above and ARA in patients with left-side IE.

### Secondary endpoints

To compare major in-hospital complications in patients with and without ARA.

### Statistical methods

All data were analyzed using the SPSS version 24 statistical software and R statistical package version 3.5.1, with a two-tailed *p* value < 0.05 indicating statistical significance. Normally distributed numerical values were reported as mean ± standard deviation (SD). For variables with a skewed distribution, data were expressed as a median and inter-quartile range. Qualitative variables were presented as counts and percentages. Comparisons of continuous data between groups were made using the two-sample *t* test or the Mann-Whitney *U* test as appropriate. The chi-square test or Fisher’s test were used to make between-group comparisons as appropriate. Statistically significant variables on univariate analysis were entered in a stepwise multivariable logistic regression analysis to determine predictors of the abscess.

## Results

Our study included 285 patients with left-sided infective endocarditis. There were 152 patients with aortic valve IE representing 53.3% of the total number of patients. ARA was detected in 61 patients representing 21.4% of the whole study population and 40.1% of aortic valve IE patients.

**Fig. 2:**
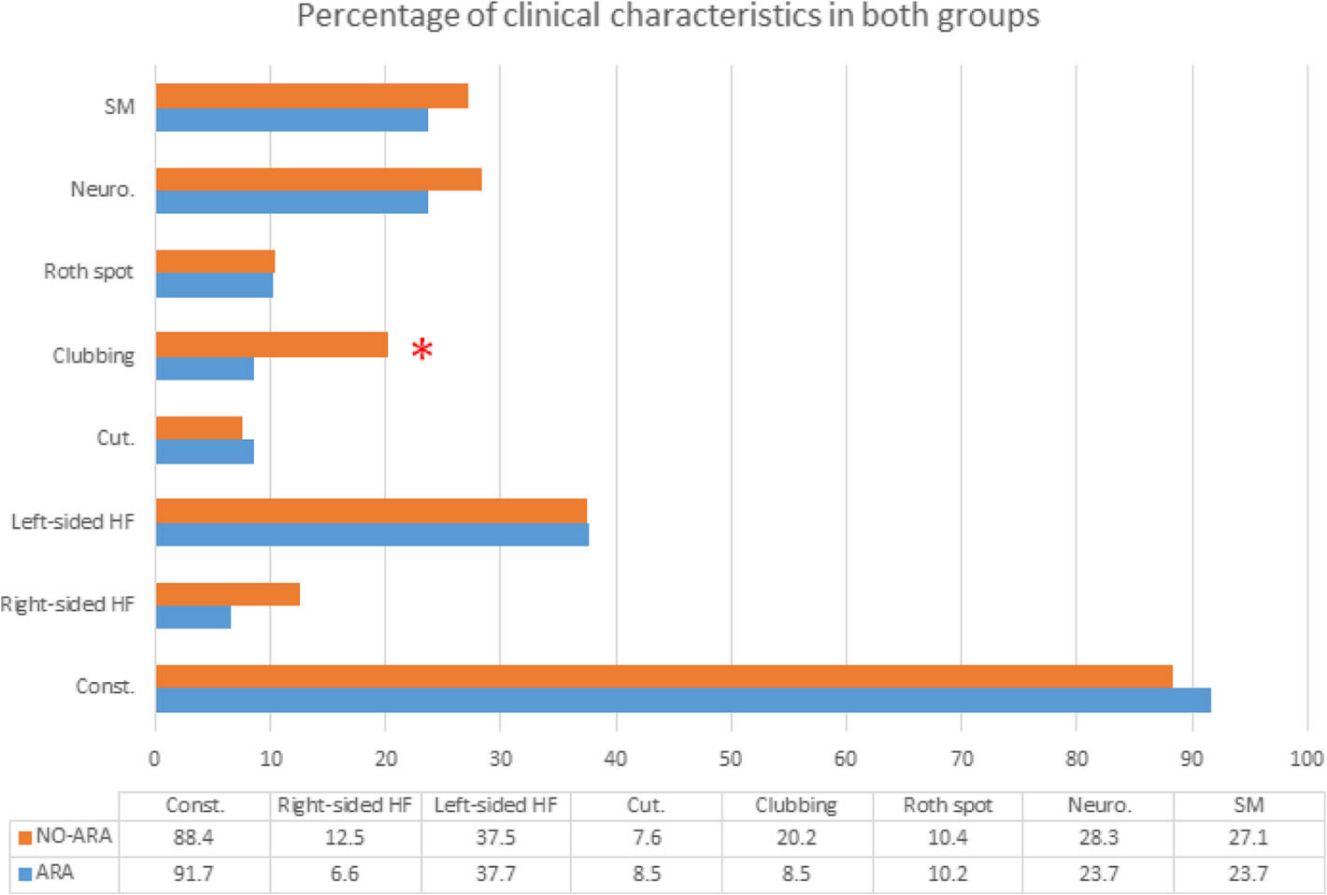
Percentage of clinical findings in both groups. ARA, aortic root abscess; Const., constitutional manifestations; Cut., cutaneous manifestations; HF, heart failure; Neuro., neurological manifestations; NO-ARA, no aortic root abscess; SM, splenomegaly. *p *= 0.04*

Table 1 shows the baseline demographic and underlying heart diseases of both groups. Male gender, prosthetic valve, congenital heart diseases (bicuspid aortic valve, subaortic membrane, and ventricular septal defects), and prior infective endocarditis were significantly higher in the ARA group. Regarding symptoms and physical signs, the NO-ARA group showed an increased incidence of clubbing compared with the ARA group (20% vs. 8.5%). The rest of the symptoms and signs were not different between both groups, as shown in Fig. 2.

**Table 1:** Demographic, clinical characteristics, and laboratory data of the patients with and without aortic root abscess, median ± IQR or *N* (%)

	ARA (*N* = 61)	NO-ARA (*N* = 224)	*p* value
Age	31 (24, 40.5)	32 (24,43)	0.36
Male gender	42 (68.9%)	122 (54.5%)	**0.04**
Underlying cardiac conditions			
RHD	27 (45.0%)	121 (54.3%)	0.20
Mechanical prosthesis	31 (50.8%)	40 (17.9%)	**< 0.001**
Congenital heart disease	11 (18.3%)	16 (7.1%)	**0.009**
Bicuspid aortic valve	8 (13.1%)	9 (4.0%)	**0.01**
Prior IE	8 (13.3%)	8 (3.6%)	**0.008**
Degenerative heart disease	4 (6.6%)	23 (10.4%)	0.37
IV drug abuse	0 (0%)	7 (3.1%)	0.35
Normal Heart	1 (1.6%)	17 (7.6%)	0.14
Comorbidities			
DM	6 (9.8%)	15 (6.7%)	0.41
CKD	4 (6.6%)	22 (9.8%)	0.43
Hemodialysis	3 (4.9%)	8 (3.6%)	0.70
Chronic steroid use	0 (0%)	12 (5.4%)	0.08
Chronic hepatic disease	1 (1.6%)	10 (4.5%)	0.47
Collagen vascular disease	0 (0%)	6 (6.7%)	0.35
Clinical manifestations			
Duration of symptoms (Days)	30 (14, 90)	28 (14, 84)	0.77
Temperature (Celsius)	38 (37, 39)	38 (37.5, 39)	0.82
Heart rate (bpm)	100 (96,120)	100 (90,110)	0.11
Laboratory data			
Anemia	52 (94.5%)	199 (93.9%)	0.999
Minimum Hemoglobin (gm/dL).	8.9 (7.5, 10)	9.0 (7.7, 10.2)	0.61
Leukocytosis	39 (68.4%)	137 (65.2%)	0.65
TLC (*10^3^/cc)	13 (10.1, 17.3)	13 (9, 18.4)	0.96
Initial Creatinine (mg/dl)	0.9 (0.7, 1.2)	0.9 (0.7, 1.2)	0.87
CRP (mg/L)	120 (48, 183)	77 (37, 130)	**0.03**
Antibiotic use before referral	**67.8%**	**70.5%**	**0.69**
Surgical management	44(73.3%)	121(66.5%)	0.32

*ARA* aortic root abscess, *CKD* chronic kidney disease, *CRP* C-reactive protein, *DM* diabetes mellitus, *IE* infective endocarditis, *RHD* rheumatic heart disease, *TLC* total leucocytic count

The laboratory data of both groups are shown in Table 1. We found that the CRP level was higher in the ARA group as compared with the NO-ARA group. The rest of the studied labs were not different between both groups.

**Fig. 3:**
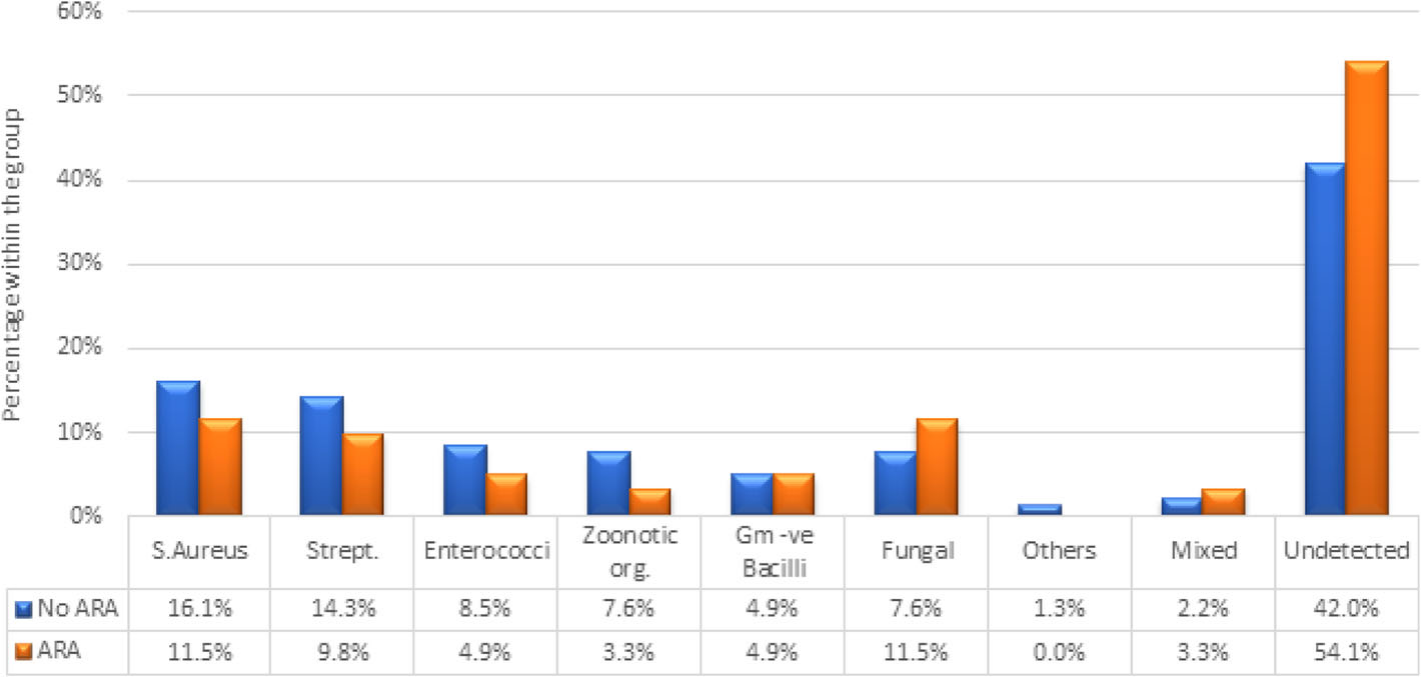
Causative organisms in ARA and No-ARA groups

Different microorganisms, as detected by blood cultures and serological tests, are shown in Fig. 3. There was a trend towards an increased incidence of undetected organisms in the ARA group as compared to the NO-ARA group (54.1% vs. 42.0%, *p* = 0.09). Antibiotic use before the referral showed no statistically significant difference between both groups (67.8% vs. 70.5%, *p* = 0.69).

**Fig. 4:**
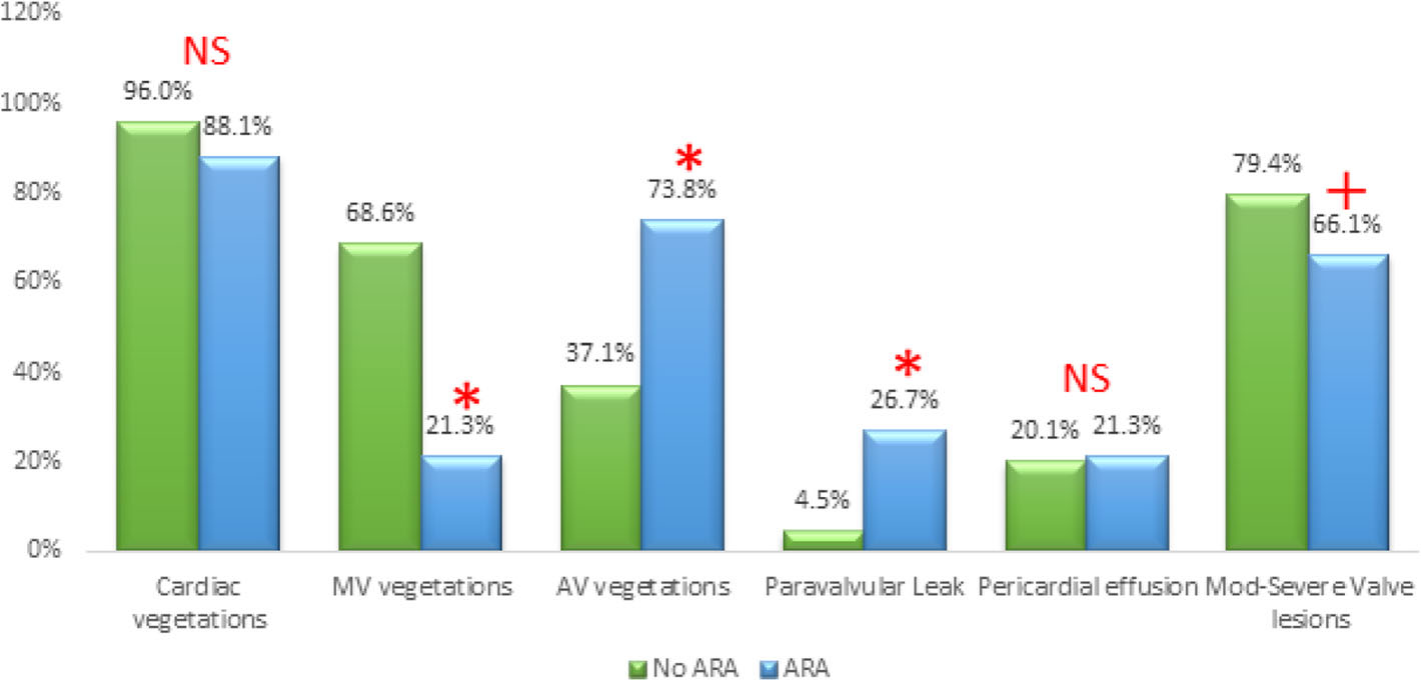
Echocardiographic findings in ARA and NO-ARA groups (NS, non-significant; *p < 0.001, ^+^*p* = 0.03)

Figure 4 shows the echocardiographic data of both groups. Overall, the vegetations were less seen in the ARA group. Reviewing the vegetations by site, the ARA group showed less mitral valve vegetations, considerably higher aortic valve vegetations, higher paravalvular leakage, and lower incidence of moderate to severe valvular regurgitant lesions. Mitral vegetations were detected in 58.5% of the whole study patients (48.2% as isolated mitral vegetations and 10.3% as combined mitral and aortic vegetations). Isolated mitral vegetations were significantly less in the ARA group (13.1% vs. 57.8%, *p* < 0.001), while there is no significant difference between both groups regarding the combined mitral and aortic vegetations (8.2% vs. 10.7%, *p* = 0.56).

Multivariate logistic regression analysis was done for the previously statistically significant data. Mechanical prosthesis, undetected organism, aortic vegetations, and paravalvular leakage were the most significant independent predictors of ARA, while the chances of ARA were lower with mitral valve vegetations as shown in Table 2.

**Table 2:** Logistic regression analysis for significant variables associated with ARA

	OR	95% CI	*p* value
Aortic PVL	3.9	1.2–13.0	0.03
Mechanical prosthesis	3.6	1.5–8.7	0.005
Aortic vegetations	3.0	1.2–8.0	0.02
Undetected organism	2.3	1.1–4.6	0.02
Mitral vegetations	0.2	0.08–0.5	0.001

*CI* confidence interval, *OR* odds ratio, *PVL* paravalvular leakage

Regarding complications, including in-hospital all-cause mortality, there were no statistically significant differences between both groups, as shown in Table 3. Similar proportions of patients underwent surgery in both groups. There was no significant difference between surgically managed patients in both groups regarding in-hospital mortality and major complications, as shown in Table 4.

**Table 3:** Major complications in ARA and NO-ARA groups, *N* (%)

Event	ARA	NO-ARA	*p* value
In-hospital mortality	22 (36.1 %)	67 (29.9%)	0.36
Heart failure Fc III/IV	28 (45.9%)	113 (50.4%)	0.53
Sepsis	15 (24.6%)	39 (17.4%)	0.21
Peripheral embolization	22 (36.1%)	74 (33.0%)	0.66
Splenic infarction	7 (11.5%)	35 (15.6%)	0.42
Mycotic aneurysms	6 (10.0%)	17 (7.7%)	0.60
Cerebral embolization	18 (29.5%)	66 (29.5%)	0.995
All embolization	32 (52.2%)	121 (54.0%)	0.83
Acute kidney injury	22 (36.1%)	64 (28.6%)	0.26
Dialysis	2 (3.4%)	11 (5.0%)	0.999

**Table 4:** In-hospital mortality and major complications in surgically treated patients in both groups, *N* (%)

	ARA (*N* = 44)	NO-ARA (*N* = 121)	*p* value
In-hospital mortality	13 (29.5%)	30 (24.8%)	0.54
Major complications	31 (70.5%)	86 (71.1%)	0.94

## Discussion

In this study, we reviewed the clinical, laboratory, and echocardiographic data of patients with infective endocarditis associated with ARA. We reported several clinical (e.g., mechanical prosthesis), laboratory (e.g., higher CRP), and echocardiographic (e.g., aortic vegetations) variables that were significantly higher in ARA patients. The odds of ARA were higher in patients with a mechanical prosthesis, aortic vegetations, aortic paravalvular leakage, and undetected organisms and lower in those with mitral valve vegetations.

The prevalence of ARA was variable in past studies. An earlier report by John et al. detected ARA in 32 out of 50 patients with aortic IE (46%) from 1982 to 1988 [6]. In Leung et al., ARA was detected in 32% of patients (11 out of 34 patients with aortic IE) in the period from 1989 to 1993 [17]. Anguera et al. identified ARA in 201 patients out of 2055 native aortic valve IE (9.8%) in a retrospective analysis from 16 referral hospitals from 1992 to 2003 [18]. In the same issue, Anguera et al. reported ARA in 150 patients out of more than 872 prosthetic aortic valve IE (17%) from the same registry [19]. Graupner et al. detected a higher prevalence of ARA in 37% of aortic IE patients (78 out of 211 patients from 1996 to 2000). In the TAVI era, prosthetic valve endocarditis was detected in 103 out of 4336 patients. Among these 103 patients, ARA was detected in 12 patients (11.9%) [20]. Our study reported ARA in 21.4% of patients with left-sided IE and 40.1% of aortic valve IE. Late presentation and use of inappropriate antibiotics could explain the high percentage in this report, which reflects the current situation in Egypt.

The median age of the study population was 31.5 years (IQR; 24, 42.2), which is much less than the worldwide reported age of ARA patients. In a recent meta-analysis, the age of ARA patients ranged from 37 to 62 years [21]. The young age of our patients reflects the type of the underlying heart disease in our study, being rheumatic or congenital heart disease in the majority of cases and degenerative heart disease in only 9.5% of cases. Age was not statistically different between both groups (ARA and NO-ARA groups), but the male gender was more common in the ARA group. Other reports for aortic root abscess also showed a trend of more incidence of ARA in male patients [9, 21].

Comorbid conditions such as diabetes mellitus and renal and hepatic diseases were similar in both groups. There was a trend of higher chronic steroid use in the NO-ARA group. Most of the patients on steroid therapy (11 patients) had mitral valve endocarditis, and only one patient had aortic and mitral valve endocarditis without abscess development. However, it is improper to assume any protective role of steroids from aortic root abscess.

We found that prosthetic mechanical valve and congenital heart disease were significantly higher in the ARA group. The prosthesis was found to be an independent predictor for ARA in previous studies [22]. The bicuspid aortic valve was the most common congenital heart disease in our study population and was significantly higher in the ARA group. Kiyota et al. [23] has shown that the bicuspid aortic valve is associated with an increased incidence of IE and ARA as compared with the trileaflet valve.

Clubbing was seen more in the NO-ARA group. This could be explained by the fact that clubbing needs some time to develop, which is not the case for IE with ARA, which is usually an aggressive, rapidly developing infection. CRP levels were higher in the ARA group. This finding could be explained by the more extensive damage and the more aggressive nature of ARA. However, we did not find any previous reports describing the above two findings.

There was an increased likelihood of microorganisms’ un-detection in our study population (i.e., negative blood cultures and serology) that may be attributed to increased use of antibiotics before referral. This microorganism un-detection was numerically higher in the ARA group compared with the No-ARA group (54.1% vs. 42%, *p* = 0.09). On the other hand, the detected microorganisms were not different between both groups. *Staphylococcus aureus* and fungal infections represent the most commonly detected organisms in the ARA group (each occurred in 11.5% of ARA patients). *Staphylococcus aureus* was commonly found in ARA patients in previous studies [24, 25].

Regarding the complications, there was no statistically significant difference between both groups, including all-cause in-hospital mortality. These results contrast previous data that showed a worse outcome of IE complicated with aortic root abscess [26, 27]. The relatively late presentation and delayed surgical intervention in both groups could be the cause of the comparable outcome.

In our study, the ARA group showed more aortic valve vegetations and less mitral valve vegetations. This finding seems reasonable in a study of aortic root abscess and not mitral ring abscess. However, this may raise a question regarding the well-known site for aortic root abscess, which is the aortomitral continuity. Forteza et al. operated upon 26 patients with aortic valve IE and intervalvular fibrous body abscess representing 10.6% of the patients with aortic valve IE [28]. Our study suggests that the spread from aortic valve endocarditis is the original site of the ARA that can extend into aortomitral continuity, rather than perivalvular infection in mitral valve endocarditis.

The paravalvular leak was significantly higher in the ARA group. The paravalvular leak is a common finding in prosthetic valve endocarditis. Anguera et al. detected moderate to severe aortic paravalvular leak in 45% of patients with prosthetic valve IE. Prosthetic valve IE usually begins as periannulitis and then spreads to adjacent tissues causing an abscess and can lead to a paravalvular leak [19]. The presence of moderate to severe regurgitant valve lesion was found to be lower in the ARA as compared to the NO-ARA group. This result can be explained in two aspects. First, all implanted prostheses in our study population were mechanical, where paravalvular leakage is much more common than transvalvular regurgitation. Second, perhaps the original site of the infection in ARA is more eccentric at the annulus more than the valve itself, favoring the spread of infection to adjacent tissues causing periannular complications rather than destroying the valve itself.

Multivariate analysis of the above variables showed that the presence of mechanical prosthesis and paravalvular leakage were the most independent predictors of ARA (OR 3.7 and 3.9, respectively). The presence of mechanical prosthesis also seems to be associated with an increased risk of development of ARA. This result highlights the importance of appropriate perioperative sterilization and disinfection, as well as the necessity of proper hygiene in patients with prosthetic valves. The presence of paravalvular leakage of any degree in the presence of clinical suspicion should be alarming to the possibility of ARA. All required investigations such as transesophageal echocardiography and CT aortography should be done to exclude ARA. Other independent predictors were aortic valve vegetations and undetected organisms. Both variables suggest the presence of the aggressive nature of infection that leads to this severe annular complication. ARA was less seen in the presence of mitral valve vegetations.

Studies reporting the predictors of ARA are scarce and relatively old. Omari et al. reported aortic valve infection and intravenous drug abuse as the most independent predictors of ARA in patients with native aortic valve IE [24]. Later, Blumberg et al. identified a new atrioventricular or bundle branch block as the only significant correlation [29]. In another study, the most common risk factors for paravalvular infection were prosthetic valve, aortic valve infection, and coagulase-negative staphylococci [30]. We could not find any recent studies highlighting the ARA predictors.

The latest trials of ARA focused on the outcome of surgical procedures. A meta-analysis by Chen et al. [21] reviewed seven surgical trials of ARA, comparing the results of aortic root replacement vs. aortic valve replacement. There was no difference between both procedures on 30-day follow-up; however, aortic root replacement was associated with a 50% reduction of the rate of reoperation on 1-year follow-up. Kirali et al. [31] showed surgical outcomes in 27 patients with ARA. The mean duration of follow-up was 6.8 ± 3.7 years. In-hospital mortality was 22.2%, which was lower than in-hospital mortality reported in our study (36.1%). Mean 1-, 5-, and 10-year survival were 70.2%, 62.2%, and 59.2%, respectively. Sultan et al. [32] studied the use of aortic homograft in 138 ARA patients with relatively not high surgical mortality (12.3%). However, 5-year mortality was again high (43%). Yang et al. [33] showed similar results in 179 patients with an operative mortality of 8.4% and 10-year mortality of 59%. So, despite the good immediate surgical outcome in ARA patients, this complication carries an increased risk of mortality on long-term follow-up.

There are some limitations to our study. First, it was an observational study, being limited by the lack of local resources and expertise. Second, underlying cardiovascular conditions, time to presentation, the causative microorganisms, and antibiotic regimen protocols may differ between different countries. Third, the relatively small number of ARA patients is considered a limitation. However, the majority of previous studies reported their results based on a similar number of patients due to the slow recruitment of ARA in clinical studies. Finally, the significant variables by multivariate analysis in our study showed a wide confidence interval. The leading cause of this wide CI was the limited sample size. The confidence intervals of odds ratios were also wide in the previous ARA studies. In Leontyev et al., one of the largest studies of surgical treatment of ARA with 172 patients, all independent predictors of mortality showed wide CI (e.g., sepsis had OR 3.6 with 95% CI 1.2–10.7) [34]. Increasing sample size could lead to a narrower confidence interval; however, the recruitment of a large number of ARA patients into a clinical study still represents a challenge.

## Conclusion

Our study showed that ARA represents a common complication in patients with left-sided IE. Mechanical prosthesis, aortic paravalvular leakage, aortic vegetations, and undetected organisms were the most independent variables associated with ARA. On the other hand, the mitral valve IE was significantly lower in patients with ARA. The presence of these variables in the context of IE should be alarming to the increased risk of ARA, and the clinician should use different imaging modalities (TEE, CT aortography, PET scan) to exclude ARA as appropriate. We recommend future multicenter research to elucidate the predictors of ARA and to evaluate the long-term outcome in ARA patients.

## Data Availability

The dataset supporting the results and conclusions of this article will be available from the corresponding author on request.

## Abbreviations

ARA: Aortic root abscess
CI: Confidence interval
CKD: Chronic kidney disease
CRP: C-reactive protein
DM: Diabetes mellitus
HF: Heart failure
IE: Infective endocarditis
NO-ARA: No aortic root abscess
NYHA Fc: New York Heart Association functional class
PVL: Paravalvular leak
OR: Odds ratio
RHD: Rheumatic heart disease
SOFA: Sepsis-related Organ Failure Assessment
TEE: Transesophageal echocardiography
TLC: Total leukocytic count
TTE: Transthoracic echocardiography
